# Idiopathic Intracranial Hypertension Papillopathy due to Hormonal Changes during Pregnancy

**DOI:** 10.1155/2023/6688445

**Published:** 2023-07-11

**Authors:** Marco Mafrici, Francesca Tona, Serena Fragiotta, Umberto Lorenzi, Lorenzo Gitto, Laura Toscani

**Affiliations:** ^1^Department of Ophthalmology, Ales-Cevennes Hospital, Ales, France; ^2^Imperial College NHS Healthcare Trust, Charing Cross Hospital, London, UK; ^3^NESMOS Department, Ophthalmology Unit, St. Andrea Hospital, University of Rome “La Sapienza”, Rome, Italy; ^4^Department of Ophthalmology, Charles-Nicolle Hospital, Rouen, France; ^5^Cook County Medical Examiner's Office, Chicago, Illinois, USA; ^6^Department of Anesthesiology and Intensive Care, CTO Andrea Alesini Hospital, Roma, Italy

## Abstract

**Background:**

The underlying mechanisms of papilledema associated with intracranial hypertension remain unclear. A case of bilateral papillary edema in a patient with chronic idiopathic intracranial hypertension who was asymptomatic during her two pregnancies is reported. *Case Presentation*. A 19-year-old Caucasian female, in her third month of pregnancy, complained of difficulties with close reading. The patient's visual acuity was 20/20 on the Snellen chart and improved with a 0.50 D correction in both eyes. Near vision and slit lamp examinations revealed normal findings bilaterally. However, a fundus examination showed bilateral papillary edema without evidence of hemorrhages or neovascularization. Blood tests were unremarkable, except for a slight increase in C-reactive protein levels. The patient had a prepregnancy weight of 63 kilograms, with a BMI of 24.91 kg/m^2^. Magnetic resonance imaging of the brain revealed features consistent with chronic idiopathic intracranial hypertension, which resolved after delivery. Two and a half years later, during a subsequent pregnancy, the patient experienced a recurrence of bilateral papillary edema due to the IIH. It was managed similarly as the first occurrence, resulting in bilateral anatomical and functional recovery. Recent research revealed that, during pregnancy, hormones interact with the central nervous system, leading to an increase in the size of neurons which could potentially result in intracranial hypertension.

**Conclusions:**

The influence of hormonal fluctuations during pregnancy on the development of transient central nervous system abnormalities in individuals with chronic intracranial hypertension, leading to papillary edema, remains a matter of debate.

## 1. Introduction

The pathophysiology behind optic disc edema in cases of idiopathic intracranial hypertension (IIH) is still not fully understood. This condition was first reported in 1853 by Türck [1], but since then, multiple conflicting theories have been proposed to explain its development. One theory, suggested by Von Graefe, posits that optic disc edema arises due to venous stasis caused by compression of the central retinal vein where it exits the optic nerve and enters the optic nerve's dural sheath [2]. Another theory, proposed by Ernest and Potts, suggests that increased intracranial pressure results in compression of optic nerve fibers in the prelaminar region of the lamina cribrosa, leading to damage of axonal flow and swelling of axonal fibers, ultimately resulting in edema [3]. A more recent theory, put forth by Hayreh, proposes that optic disc edema in IIH occurs in two phases: the first phase is characterized by impaired axoplasmic flow, and the second phase involves impaired optic nerve microcirculation [4].

Papillary edema, which is a manifestation of optic nerve head impairment, can result from various underlying pathologies. A thorough differential diagnosis is crucial to rule out potential causes such as autoimmune inflammatory diseases (sarcoidosis and Horton's disease), infectious diseases (Lyme disease and tuberculosis), tumor infiltrations (leukemia), vasculopathy, and diabetic papillopathy [5]. The clinical diagnosis of papillary edema associated with intracranial hypertension requires the presence of subjective symptoms such as headache, nausea, vomiting, and objective radiological and/or objective laboratory findings, such as magnetic resonance imaging (MRI) and lumbar puncture with cerebrospinal fluid (CSF) analysis [6–8].

Optical coherence tomography (OCT) and retinal fluorescein angiography are critical diagnostic tools in the evaluation of papillary edema. These investigations enable the monitoring of vascular and anatomical changes in the optic nerve and retina.

Several researches on intracranial hypertension during pregnancy have been published in the literature. Some of these studies have indicated a potential correlation between increased body weight and a higher risk of developing IIH during pregnancy [9–11]. Recent research conducted on laboratory animals has revealed that pregnancy can cause significant morphological and functional changes in the brain tissue and neurons of these animals [12].

In the present paper, a case of IIH-related papillary edema relapsed during two pregnancies unrelated to weight gain is reported.

This study is a retrospective observational analysis conducted at the Department of Ophthalmology at Ales-Cevennes Hospital in Ales, France. The study involved a blind review of the patient's medical records.

## 2. Case Description

A 19-year-old white female patient, at the end of the third month of her pregnancy, presented with difficulties in close reading. Her vision examination showed 20/20 using a Snellen chart, which improved with a correction of 0.50 D in both eyes. Near vision and slit lamp examinations were normal bilaterally. However, an ocular fundus examination revealed bilateral papillary edema without hemorrhages or neovascularization. No other retinal abnormalities or vitreous inflammation was detected. The patient's medical chart was reviewed by masked assessors, and she was admitted to the hospital for further investigations. Blood work showed slight increases in C-reactive protein values, but no evidence of autoimmune diseases was found. The patient's prepregnancy weight was 63 kilograms, with a BMI of 24.91 kg/m^2^. At the time of admission, her blood pressure was 120/90 mmHg, and she was 1.64 centimeters tall and weighed 65 kilograms.

**Figure 1:**
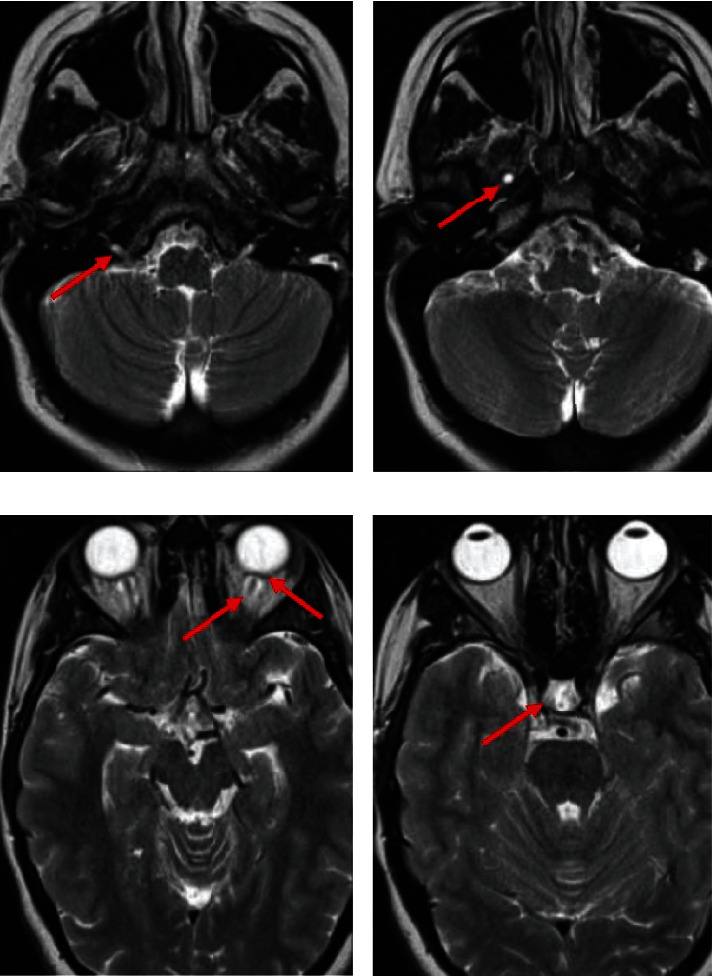
Magnetic resonance imaging findings: enlarged hypoglossal canal (a), small petrous apex meningocele (b), perioptic nerve sheath distension and flattening of the posterior ocular globe (c), and enlarged cerebrospinal fluid filled pituitary fossa (“empty sella”) (d).

After the first trimester of pregnancy, an MRI with gadolinium was conducted to exclude any secondary causes of intracranial hypertension. The imaging showed several findings, including an enlarged hypoglossal canal (Figure 1(a)), a small meningocele in the petrous apex (Figure 1(b)), perioptic nerve sheath distension, posterior ocular globe flattening (Figure 1(c)), and a slightly enlarged pituitary fossa filled with cerebrospinal fluid (“empty sella”) (Figure 1(d)).

**Figure 2:**
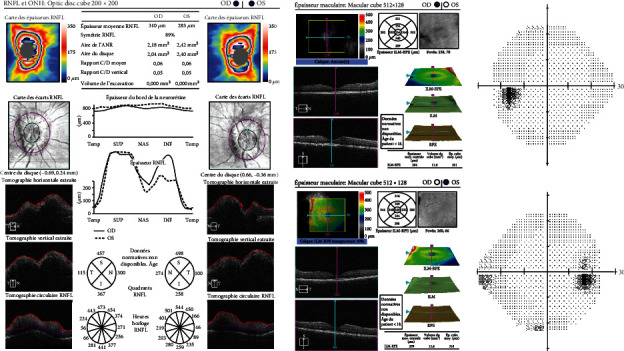
Optical coherence tomography (a, b) and visual field test (c) findings of the right and left eyes at the initial examination before delivery (first pregnancy).

Further eye exams revealed a slightly abnormal color vision test and normal visual field (Figure 2(c)). An optical coherence tomography (OCT) showed increased papillary thickness and regular macular profile bilaterally (Figures 2(a) and 2(b)).

Fluorescein angiography was planned, but the patient declined the administration of a contrast agent due to concerns related to pregnancy. Therefore, this investigation was not performed.

The patient's medical history, clinical presentation, and imaging findings suggested a diagnosis of bilateral papillopathy related to chronic intracranial hypertension (IIH) caused by pregnancy. A lumbar puncture performed shortly after delivery revealed elevated cerebrospinal fluid (CSF) with normal pressure and composition, confirming the diagnosis of IIH.

**Figure 3:**
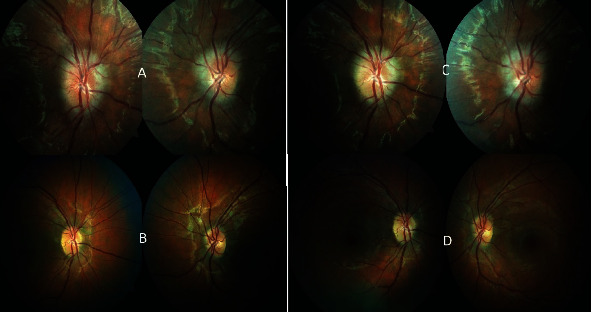
Right and left papillary edema detected during the initial examination of both eyes during the first pregnancy (A); right and left papilla with no evidence of edema 2 months after delivery (B); right and left papillary edema detected during the initial examination of both eyes during the second pregnancy (C); right and left papilla 1 month after delivery (second pregnancy) (D).

The patient's BMI increased to 29 kg/m^2^ by the end of her pregnancy. The bilateral papillary edema resolved within two months after delivery without any residual damage or drug intervention (Figure 3(B)). The patient continued to be monitored by the ophthalmology service, and during her second pregnancy, she experienced a recurrence of bilateral optic nerve edema (Figure 3(C)). A similar battery of tests was conducted, and the results were consistent with the first episode. A diagnosis of recurrent bilateral papillary edema related to chronic IIH by pregnancy was established, and the condition was managed similarly to the first episode resulting in full recovery after delivery (Figure 3(D)).

## 3. Discussion

Studies in the literature suggest a potential correlation between weight gain during pregnancy and an increased risk of intracranial hypertension and papillary edema [9–13].

In the presented case, the patient experienced only a small weight gain during the first trimester of pregnancy (approximately 2 kilograms), which would not be expected to cause intracranial hypertension and papillary edema. Additionally, the fact that the papillary edema resolved within two months after delivery despite a much greater weight gain during the entire pregnancy (approximately 8 kilograms) suggests that weight was not a significant factor in this case. A possible explanation for the absence of symptoms is that the patient may have experienced a “compensated IIH,” a state in which the pressure inside the skull is elevated but the brain can still maintain normal blood flow and function by adjusting its volume and/or the flow of cerebrospinal fluid [8].

The physiological changes that occur during pregnancy can affect various systems in the body, including the cardiovascular and endocrine systems, leading to potential complications such as gestational diabetes and eclampsia [14, 15]. However, it is unclear if these hormonal changes can also cause any biochemical, morphological, or functional changes in the central nervous system.

Recent research suggests that pregnancy-induced hormonal changes can interact with the central nervous system and cause an enlargement of neurons. A recent study using morphometric MRI on pregnant mice showed temporary changes in the brain's morphology [12]. Interestingly, the patient experienced papillary edema due to IIH only during her two pregnancies, with regression after delivery. Hormonal changes during pregnancy may lead to temporary central nervous system changes and subsequent abnormalities in cerebrospinal fluid pressure. Therefore, pregnancy could pose a potential risk for IIH, independent of weight gain during pregnancy.

This may help explain why not all women who experience significant weight gain during pregnancy develop intracranial hypertension. It is possible that hormonal changes may contribute to the development of intracranial hypertension in pregnant women, and therefore, monitoring the hormonal levels of patients before and after pregnancy may be important in addition to monitoring BMI.

Therefore, it may be important to consider hormonal changes and levels in the management of intracranial hypertension related to pregnancy, as they could impact therapeutic outcomes. Evaluation of hormonal levels before starting diuretic therapy may be beneficial in optimizing treatment effectiveness.

A limitation of our study is that it was not possible to perform lumbar puncture during pregnancy because the patient did not agree to undergo this examination during pregnancy. Additionally, it was not possible to perform contrast-enhanced exams because the patient was afraid of the potential toxicity of contrast agents.

In ophthalmology, noninvasive examinations such as OCT, angio-OCT (OCT-A) [16, 17], and recently, the ultra-widefield scanning laser ophthalmoscope (UWF-SLO) [18] plays a fundamental role.

In the future, the identification of specific biomarkers could be helpful in improving the diagnostic criteria and follow-up of papillopathy and maculopathy [19]. Moreover, the use of ultra-widefield fluorescein angiography (UWF-FA) has been introduced, which allows for fluorescein angiography testing without the need for intravenous injection and may be beneficial, particularly in pregnant patients, as it may be perceived as less impactful during the course of pregnancy [20].

## 4. Conclusions

The hormonal changes that occur during pregnancy may induce modifications in the central nervous system, which may contribute to the development of intracranial hypertension, regardless of weight gain. In such cases, papillary edema may potentially regress when hormone levels return to baseline concentration postpartum, as demonstrated in the presented case.

The identification of specific biomarkers and the use of ultra-widefield fluorescein angiography (UWF-FA) may be beneficial to effectively diagnose and treat this uncommon condition.

More research is required to comprehend the underlying pathophysiology of papillary edema associated with IIH during pregnancy, particularly regarding possible pregnancy hormone-induced alterations in the central nervous system.

## Data Availability

The data used in this current report are available from the corresponding author on reasonable request.

## Abbreviations

OCT: Optical coherence tomography
OCT-A: Optical coherence tomography angiography
UWF-SLO: Ultra-widefield scanning laser ophthalmoscope
UWF-FA: Ultra-widefield fluorescein angiography
IIH: Idiopathic intracranial hypertension
RNFL: Retinal nerve fiber layer
MRI: Magnetic resonance imaging
BCVA: Best-corrected visual acuity
PCR: Reactive protein C
VFT: Visual field test.
